# Notes on the Asylums of Italy, France, and Germany

**Published:** 1860-07-01

**Authors:** J. T. Arlidge


### Art. IV.—NOTES ON THE ASYLUMS OF ITALY, FRANCE, AND GERMANY.

By Dr. J. T. Arlidge, M.B., A.B., Lond., M.R.C.P., &c.

(*Continued from p.* 566, *vol. xii.*)

### GRENOBLE,

The capital of the department of the Isère, is situated on the banks of the river Isère, in a large and beautiful valley, overhung by bold, high, and rocky hills, and possessing great charms for the traveller in search of the beauties of natural scenery. In the course of the same valley, and some four or five miles from the town of Grenoble, is the small hamlet of St. Robert, in which the asylum is situated. The asylum buildings are not more than two

**Philosophie der Geschichte,* 1 Th., pages 193 and 163.

or three hundred yards from the public road, and at no great distance from them, on their opposite side, is the Lyons and Grenoble railway. The institution serves for the department of the Isère, and for that of the Hautes Alpes.

In the summer of 1855, the 260 patients then present were crowded in an irregularly built house, formerly a small Benedictine convent, and in some indifferent out-buildings. The house was four storeys high, but the uppermost storey, as is common in French houses, especially in provincial towns, was used only as a store-room. The separation between the two sexes was most imperfect; the sitting-rooms bare and wretched, with nothing but concrete floors, worn into holes. The bare white-washed walls, the earthen floors, the benches fastened round the walls, and the heavy fixed wooden table in the centre, together with the poor patients huddled together from want of space, almost indiscriminately or without classification, many of them ragged and disorderly in dress, and many shoeless,—made a *tout ensemble* of wretchedness fortunately not often paralleled.

Happily the misery of the day-rooms did not extend to the sleeping apartments up-stairs, which were clean and in order. The bedding was also sufficiently good, consisting of a paillasse, a flock bed, with sheets and coverlets. At least, such was the provision for the clean and quiet inmates; but the wet lay upon straw with a sheet beneath them; whilst the very refractory were condemned to the seclusion of cells, and littered in loose straw contained in a trough bedstead, overlaid by a coverlet. These cells were constructed in a small, detached building, and were certainly unfit for human habitation, their ventilation being very bad, and their walls and floors of stone. A row of four such stable-like cells was furnished for each sex, and as the two rows were only separated by a narrow court and by a low wall, the cries and noises of their poor inmates reached from one division to the other, and could not fail to promote the insane fury which the isolation in cells was resorted to with the view to suppress.

Such is a rough sketch of one of the smallest and poorest departmental asylums in France, as found in operation in 1855. Since that period, fortunately for the afflicted creatures who inhabited it, for the sake of humanity, and for the credit of the French Government, this wretched establishment has made room for one which is an ornament to the department, and admirably suited for its benevolent objects. This happy exchange has been mainly due to the untiring exertions of the director and chief physician, Dr. Evrat, in face of the determined opposition of local authorities, and of great difficulties in securing the co-operation of the central government. To M. Evrat is also due the excellent plan of the new asylum, constructed for 300 patients, which differs from that of every other in France, and presents in many respects an improvement. It is difficult to convey an accurate conception of the ground-plan of the buildings without a diagram; for, like almost all French asylums, it consists of several distinct sections or disjoined wings. But it may be described generally as a group of two-storeyed dwellings, distributed around a large square court. This court, however, is cut into two by a wide avenue extended through it, which constitutes the line of division between the section of the asylum devoted to male on the one side and to female patients on the other. In the centre of the large square court or garden are the general offices, the kitchen, dispensary stores, and the residence of the physician, and some workshops.

In advance of one end of the central avenue is the entrance gate, with rooms on either side for the chaplain and internes, and for the gate-porter, gardener, and other subordinate officers. At the opposite extremity of the same avenue is the chapel. The portion of the square on each side and at each extremity of the central avenue, is occupied by two contiguous united sections, forming a sort of double house, and having a common staircase between them. These two sections are occupied on one side the square, severally by the infirmary and by the apartments of the pensioners or private patients, and on the other by quiet industrious and by feeble demented patients.

The other two sides of the square are formed by three buildings in a line, in the rear of the central one of which, in each division, male and female respectively, is a row of six cells, and in front of it a bath-house. The middle section is appropriated to idiots, noisy and dirty patients; whilst that on one side of it is occupied by convalescent and melancholic patients, and that on the other by refractory inmates. The cells forming the detached section in the rear are used for seclusion and for violent patients, not however as constant abodes. Each cell has a small court of its own, as at the asylum at Auxerre.

On the female side the uniformity of construction is partially destroyed by the retention of portions of the old asylum. The whole edifice is built of stone and in good taste. Each section is composed of a day-room and a dining-room on the ground floor, and of a couple of dormitories on the floor above, and has behind it an airing court or garden, nearly forty feet in length by thirty in width, surrounded on its exposed sides by a sunk fence. The several sections are connected together, and with the bath-house, by a covered corridor. The officers' residence, in the middle of the central court, has three storeys; and the central of the three buildings on two sides of the square has likewise a third storey, intended for the occupation of attendants. The windows in every section are alike, and unprotected by bars or wire-work. They are formed after the fashion of French casements, in each of which are four square compartments, divided by diagonal frames intersecting at the centre.

The warming of each section is effected by a stove (furnace) placed in the basement, the heat from which enters the rooms above through several square apertures along the walls, close to the floor; whilst to serve the purpose of ventilation, other similar apertures conduct the impure air into the shaft of the furnace, which draws it off and discharges it above the roof.

Except in the case of the pensioners, and the varying but always small number of occupants of cells, all the rest of the population is disposed of by night in dormitories, each of which contains twelve beds for patients, and one for an attendant.

Such is an outline of the general disposition and arrangements of the parts of the new asylum. One evident advantage accruing from the plan is that each section faces outwards; or, in other words, its fore front looks towards the surrounding country, whilst its back front faces the large inner square court. This is certainly an improvement upon the system common in France, wherein the several sections of the two divisions are disposed in parallel lines, on either side the block of building occupied by the chief officials and the general offices, so that one section faces another on one or both sides, and is separated only by a comparatively narrow airing court. Moreover, in the asylum at Grenoble, windows have been introduced on two sides of the rooms instead of one, as is more general, whereby a greater degree of cheerfulness and more effective ventilation are secured.

Respecting the management of the asylum, a few words will suffice. About eight acres of cultivable ground surround the asylum, and give employment to a considerable number of patients. A portion is laid out as a flower garden, and the rest is set apart for the growth of wheat, vegetables, &c. Cows are kept to furnish the milk required in the establishment. There is a water-mill on the premises, which grinds the corn, and bread is made in the asylum; whilst by the exercise of the patients at their several trades, all the other requirements of the community and of the building are supplied. Among exceptional appliances possessed by this asylum, a swimming bath, constructed in the garden, for the men, may be mentioned. It is fed by a stream which runs into the Isère.

The patients partake of three meals a day; potage twice a day, and a meat dinner with vegetables. Forks and spoons are allowed, but not knives. Restraint is rarely used.

In 1855 there were 260 inmates. The rapidity of their multiplication, and the short-sightedness of most non-professional men, particularly in years gone by, is exhibited in the history of this asylum at Grenoble. The Minister of the Interior, relying on the medical reports supplied to him, wrote the Prefect of the department in 1843, to the effect that, although there then were but 80 patients in the institution, accommodation for 200 would be required after the lapse of some few years. To this statement M. le Préfet demurred, and decreed the accommodation to be provided at 100; but to the confusion of all his calculations, 208 inmates were present in the asylum so soon after as 1851.

The impediments raised by such individuals as the 'Préfet' referred to, together with the prevailing prejudices and the deficiency of information respecting the wants of the insane, and their treatment elsewhere, existing in this remote department of France (distinguished for the ignorance and superstition of its inhabitants), led to the postponement of the plan for building a new asylum, and to the production of that sad state of affairs in 1855, sketched at the commencement of this paper. The management of the asylum had then come to a dead-lock. 260 patients were crammed into a building having proper space for only 80, and unfit, as we have seen, in every essential particular, for the residence and treatment of insane people. Looking to these facts and to the circumstance that the requisite funds for the proper clothing of the patients and for the internal economy of the institution were withheld, it would be unfair to visit M. Evrat with condemnation for the sad condition of affairs found at that date. Indeed, no one who then saw him could fail to sympathize with him in the arduous struggle he had so many years sustained single-handed, to attain what he was then on the point of seeing realized—the construction of an asylum adapted as a place and an instrument of treatment.

Before closing this notice of the Grenoble Asylum, we may add, as a memorandum of its statistics, that of the 260 inmates there were 8 epileptic women and seven men; 4 paralytic women and 21 men. In the new asylum epileptics are excluded.

To the reader interested in asylum construction, we may recommend the excellent paper by M. Evrat, in the *Annales Médico-Psychologiques* for 1853, p. 177, wherein will be found the leading principles which should guide in the erection and organization of asylums tersely, but very correctly laid down.

### STEPHANSFELD.

The Asylum of Stephansfeld, one of the best-known and most lauded institutions for the insane in France, is situated in a very small hamlet nearly two miles from the Brumath station on the Paris and Strasburg Railway, and some eight miles from Strasburg. The surrounding country, for a long distance, is flat, and forms part of the wide alluvial valley of the Rhine; it is highly cultivated, intersected by numerous small streams, which, from defective drainage, produce much swampy ground, and its concomitant, intermittent fever. The immediate site of the asylum is, however, drier, and stated to be healthy, although published Reports show that its inhabitants have suffered severely in some years from ague. However, the statement made to me that ague, though endemic in the vicinity, did not visit the inhabitants of the asylum, may be assumed as true of the ordinary state of things; for the irruption of that malady among the inmates in 1846, and its sad prevalence from 1854 to 1856, are distinctly traceable first to the disturbance, in 1846, of the ground in its immediate vicinity, and to the consequent interference with the drainage, caused by the construction of the railway embankment, then to the formation of the canal between the Marne and Rhine, and subsequently to the infiltration of the waters of the canal, between the autumn of 1854 and June, 1855, through the sandy soil, into all the basement portions of the building. Indeed, the history of the prevalence of intermittent fever in the Stephansfeld Asylum, as recorded in the Report of that institution for 1855, affords an excellent illustration of the causes affecting the generation of marsh-miasma. It would be a digression from the purpose of these notes to give more detail on these matters, yet the regret may be expressed that the site of this asylum is not more healthy and more removed from the possible operation of causes which have so readily and largely affected its salubrity.

The Stephansfeld Asylum receives patients of both sexes suffering from every form of mental disorder. It is especially intended for the insane of the departments of the Bas-Rhin and Haut-Rhin, but it is also resorted to by patients from other departments and other countries. Thus, of the 849 persons treated during the year 1857, 465 belonged to the department of the Lower Rhine; 321 to that of the Upper Rhine; 41 were from other departments, or from abroad; 12 had been transferred from prisons, and 10 were soldiers.

The site of the present structure was in the first place occupied by a Commandery of the Order of St. John, but of this ancient foundation nothing remains except the two picturesque towers of the church. Subsequently, a gentleman's château was erected, which, after having served for a time as a foundling hospital, was converted to its present use in 1835. Since that period the original building has undergone successive enlargements, and an extensive, irregularly distributed structure is the result.

A description of the asylum in writing, without plans, would be well-nigh useless were it desirable, which it is not; for considering it merely an adapted building, altered and added to from time to time, without reference to an original plan, its construction cannot be referred to as a model for institutions of a similar character. Still it must be admitted that the *tout ensemble* of this asylum on approaching it from the road is cheerful, and devoid of the appearances of a place of restraint. The elevation of the old château, which is appropriated to the residence of the highest class of pensioners, is pleasing. From each extremity of it a one-storied building is extended forwards so as to enclose the large front garden on two sides; in this building are the residences of the chief officers and some of the general offices. One or more of the courts in the rear are too small and too enclosed and shaded by the surrounding buildings, and therefore ill-ventilated, liable to dampness, and dull.

The upper classes of pensioners have each, according to the rate of payment, a bedroom, or this together with a well-furnished sitting-room, and for their joint use there are besides, a drawing-room (*salle de réunion*), a dining-room, a billiard-room, and a reading-room. The casements of the rooms on the first floor open out upon a wide balcony overlooking the pleasant and well-kept front garden. This balcony is protected only by a light iron rail about four feet high, and though the patients are allowed free access to it no accident had ever occurred. A room containing some half-dozen beds is set apart for each sex of pensioners as an infirmary. The portion of the front pleasure-garden for each sex is laid out in walks, one of which is trellised over, and in each division is a sort of large summer-house (*pavillon*) wherein the patients receive the visits of their friends.

The other inmates of the asylum are disposed in different sections or divisions, according to their mental state and general condition, that is, according as they are tranquil or rather troublesome, or refractory, or epileptic and dirty, or require nursing in the infirmary. The construction of the several divisions is such that all the day-rooms are placed on the ground-floor, and the dormitories on the one and two stories above. Likewise the sitting-rooms occupy the entire width of each block of building, and have windows and an external corridor on either side. In the division for tranquil patients there are a dining-room, a sitting-room, and a school-room and library; but in the other sections a dining-room and a sitting-room, or one day-room only is found. The walls of some of the sitting-rooms are decorated with landscapes for the purpose of arousing the attention and of amusing the occupants. The windows are undefended by bars or wire, and many of the doors have their upper half glazed. The refractory and epileptic sleep on the ground-floor in small single rooms; sometimes their beds are placed upon the floor to avoid accidents from violence or from falling out of bed. There is also a row of seven cells for each sex, with a day-room at one extremity intended principally as places for seclusion for the most refractory patients. These cells look too much like cages for wild beasts; they have a door back and front, according to a venerable French plan for entering upon a patient unawares at one door while his attention is engaged at the other, and through the vertical wooden bars of the window, and of the upper half of one door, the excited occupant is gazed upon much as an imprisoned wild beast is in its den. Another peculiar provision is met with in these cells, viz., an inspection aperture in the ceiling, through which a person in a loft above may see what is going on below, avoid encountering the patient, and at the same time escape observation. Dr. Webster, we perceive, has spoken of these cells and their inspection openings with approbation when he wrote his account of them in 1852. He would, however, probably materially modify his opinion at the present time, when experience, guided by humane considerations and by a better conception of the phenomena of insanity, has led in a great measure to the disuse of seclusion in this country, just as previous experience resulted in the abolition of mechanical restraint. Not but that seclusion is at times salutary; it is only the very frequent recourse to it which is objectionable, such as prevailed for a time when the disuse of mechanical coercion was progressing in the country, and some half-way step between restraint and the liberty of the present day was felt to be necessary. Moreover, in the management of the insane, all expedients to surprise, to take the patient at a disadvantage, to peep at him unperceived—such as are the double door and the hole in the ceiling, in the cells under remark—proceed on an erroneous principle, by treating him as an exceptional being, the subject of suspicion and of fear, and will find no approval in the eyes of those who best understand the treatment of lunatics.

Restraint by means of the camisole is employed at Stephansfeld for destructive and violent patients; and, at night only, for those given to self-abuse. At times, also, patients are fastened in chairs to prevent their moving about or falling forward, by means of straps.

The bedsteads are mostly of wood; those of the pensioners of mahogany. The mattresses are stuffed with horse-hair, and in the case of dirty patients are made in three portions, so that the central one, which by its position is chiefly or alone soiled, may readily be removed and replaced by another without disturbance of the whole mattress.

There is a bath-house for each sex, divided within into several compartments, in each of which is a copper bath furnished with a weavy wooden lid, so made as to be extended at will either over the whole or only one-half of the top of the bath. In front of a bench placed along the wall of the space or corridor of the bath-room are placed at short distances some three or four copper foot-baths, which are filled with hot water through a pipe opening at their bottom.

Their utility is, however, rather apparent than real, and we believe these foot-baths are little used; for it appears to an ordinary observer to be making a great matter of a little affair to make such a provision of foot-baths in a bath-room, and to bring patients to them from other parts of the building, when it is considered how portable foot-baths are, and how much more desirable it is when they are wanted to carry them to the patient than him to them.

Prolonged baths are employed in acute cases of mania, and also in melancholia, usually with the addition of a small thread of water falling on the head of the patient during his immersion in the bath. It is under such circumstances especially that the lids are fixed on the top of the baths, in order to prevent the patients leaving them. The head is defended by a bathing-cap when subjected to the stream of cold water, or occasionally a sponge is placed upon it. The douche is used not medically, but as a means of repression, particularly when food is obstinately refused.

All the courts are planted with trees and flowers; they are enclosed by walls, owing to the proximity of public paths and roads. In one court, on each side of the asylum, an artificial mound is raised so as to overlook the walls and to afford a view of the surrounding country. A large tract of arable land belongs to the asylum, of which some eight or ten acres are cultivated as a garden, partly as a flower, and partly as a kitchen-garden. A curious and beautiful sun-dial—a remnant of the property of the Knights Templars—stands in the garden, and is interesting as a work of art.

On the male side, separated only by a wall from the airing-courts, are some farm-buildings; a barn and granary, stables, a cow-house and piggery. This last must by its proximity to the asylum be a nuisance, particularly in hot weather, and it is proposed to remove it farther away.

The government of the institution is entrusted to the Prefect of the Department of the Bas-Rhin, in which it is situated, and to a commission of gentlemen appointed by him. The general management is delegated to the director, who is not required to be a medical man, whilst the medical organization and treatment are entrusted to a resident physician. The former officer, however, ranks first, and besides his superior position, his immediate relation and communication with the governing commission and the prefect give him a preponderating power in all that relates to the management of the institution. This division of an authority which can only be satisfactorily held in the hands of a single person, must be fraught with many evils and be prejudicial to the welfare of the establishment. The physician is assisted by two "internes" and an apothecary. On the male side all the attendants are hired servants, with a chief attendant at their head; on the female side, the duties of the house are performed by the Sisters of Charity of St. Vincent de Paul, under the direction of a resident superior. The "lingerie," or linen store, the laundry, and the kitchen, and the infirmaries, are also under the management of the "sœurs," assisted by patients, and a few of the fraternity assist at meal-time on the male side.

The religious interests of the inmates are committed to three chaplains, one of whom is minister for the Roman Catholics, another for the Protestants, and the third for the Jews. The same spirit of religious toleration has provided separate chapels for the Roman Catholics and Protestants; the former much the larger, and fitted and decorated as other churches of that sect; the latter a large plain room, with nothing of an ecclesiastical character about it except a pulpit or desk. The chaplains are allowed free access to the wards, but their ministrations to particular patients are under the guidance of the physician.

The very laudable endeavour to instruct the poor inmates of the asylum is assiduously carried out. On the male side there is a specially hired instructor and a separate school-room, furnished with tables, writing and drawing materials, slates, and books, and having its walls hung with maps, diagrams, and tables. On the female side one of the "sisters" superintends the teaching. Mutual instruction is encouraged; drawing, reading, and singing in classes carried out as far as possible, whilst information and amusement are afforded by lectures and concerts. The reports made of the systematic efforts to educate and improve the mental powers of the patients are very encouraging, and make particular mention of the occasional speedy benefit effected on the minds of some of the insane by the sight of what is going on around them, and of the questions addressed to them.

Three meals are allowed daily. For the poor the first meal or breakfast is composed of soup containing bread; the dinner of bread, soup, legumes, and on most days meat; the supper either of soup, as in the morning, or of bread with cheese and salad or legumes. Those patients engaged with work have a lunch between breakfast and dinner, and are allowed a little wine. Snuff and tobacco are also granted to those habituated to their use. The pensioners have meat twice a day, a greater variety in food, and about half a pint of wine. The highest class of pensioners pays no more than £60 per annum, and for this each individual is entitled to an excellent bed-room and sitting-room, and the services of a special attendant.

The moral treatment of the insane is well understood at Stephansfeld, and for the most part efficiently carried out. There are doubtless blemishes in its management; the neatness and cleanliness of the rooms and patients are susceptible of improvement, and the dealing with the refractory as rather objects of fear to be put out of the way, or coerced and punished for the effects of their delirium, is a proceeding based on a faulty appreciation of their real condition. However, we have no wish to assume the office of censors in regard to the establishment under notice, in which so much that is excellent may be discovered in its management, and where we may hope that the same enlightened and humane principles which have prospered so far may still further develope and yield still more beneficial results.

The value of employment, particularly in the open air, is very fully appreciated. The large tract of ground belonging to the asylum affords scope for it, and in order to stimulate still more the readiness of the patients to employ themselves, the former physician, M. Roederer, instituted a system of rewards, whereby every labourer acquired a material interest in his work. The labourers were divided into five classes, according to the amount and excellence of the work executed; the work was valued monthly, and its value divided, allowing one-half to the worker in extra articles of diet and in money, and transferring the other to the institution, which devoted it chiefly to a fund for relieving patients on and after their discharge. Field and garden work is most promoted among the male inmates, and few are occupied with in-door trades; indeed no special workshops are yet erected.

No particular days are fixed for the visits of friends. Letters are allowed after an examination of their contents. The patients are permitted to walk out in the neighbourhood; the pensioners daily or nearly so; the pauper inmates on Sundays and Feast-days. The great fête days are also more especially selected for their *réunions*, and concerts, and for indulgence in various games. Dramatic performances have been occasionally held, but dancing is not practised.

The extended Notes by Dr. Webster on this asylum, in the Fifth Volume of this journal, have, to avoid repetition, induced me to curtail my remarks on its structure and management, as I would wish this present paper to have rather the character of a supplement to his able account, particularly to the statistical facts collected by him, and respecting which we have much more recent information.

The rapid accumulation of patients in the lunatic asylums of this country, which has caused so much alarm as a seeming evidence of the great prevalence and increase of insanity in the population, has its parallel in the history of the asylums of France, as the statistics of Stephansfeld afford a striking instance. From the date of the opening of this establishment, on November 1st, 1835, to December 1st, 1855, a period of twenty years, 2440 patients were admitted, besides 353 re-admitted, forming a total of 2793 admissions. Of the 2440, 1267 were males and 1175 females, showing an excess of 92, or of nearly one-twelfth the former above the latter.

The following table illustrates the progressive increase of admissions. The sudden rise in the number in 1841, is due to the transmission in that year of seventy-five insane persons from the Maréville Asylum, the greater number of whom belonged to the Department of the Moselle:

Admissions.—Total, 2793.

| | | | | | | | | |
|---|---|---|---|---|---|---|---|---|
| Original population in 1835, 96. | 1836 | 49 | 1841 | 191 | 1846 | 135 | 1851 | 150 |
| | 1837 | 77 | 1842 | 98 | 1847 | 135 | 1852 | 205 |
| | 1838 | 82 | 1843 | 91 | 1848 | 155 | 1853 | 203 |
| | 1839 | 87 | 1844 | 104 | 1849 | 123 | 1854 | 231 |
| | 1840 | 90 | 1845 | 120 | 1850 | 155 | 1855 | 222 |
| | | 385 | | 604 | | 703 | | 1011 |

On comparing the sum of admissions in each of the four quinquennial periods, and taking that of the first period as a standard, it will be seen that the total of the second period is more than half as much again; of the third, nearly double, and that of the fourth, not far from treble of it. On the other hand, the discharges, including deaths, have not at all followed the same progressive ratio as this table proves:—

| | Admissions. | Discharges. | Remaining. |
|---|---|---|---|
| 1836 to 1840 | 385 | 253 | 132 |
| 1841 „ 1845 | 604 | 497 | 107 |
| 1845 „ 1850 | 703 | 618 | 85 |
| 1850 „ 1855 | 1011 | 829 | 182 |
| | | Total accumulation | 506 |

This sum, added to the original population in 1853, gives 596, the number of inmates existing on 31st December, 1855.

The ratio of recoveries to admissions in each quinquennial period cited was:—for the first, 24 per cent.;—for the second, 28 per cent.;—for the third, 25 per cent.; and for the fourth, 24 per cent. This unfavourable result—the decreasing rate of recoveries—is attributed to the transmission of so many incurables, who constituted in 1850, 35 per cent. of the entire number admitted, and in 1855, nearly 60 per cent. To obviate the crowding of the asylum, therefore, with incurable cases, the prefect of the department proposed in 1856 to restrict the admission of insane persons into the institution to two classes:—1, dangerous lunatics; 2, those in whom there were reasonable prospects of cure. This scheme may serve very well the object of limiting the extension of the Stephansfeld Asylum as a refuge for incurables, yet the success attending its operation must be accounted a positive evil, by depriving a very large proportion of the mentally afflicted of the necessary care of an asylum, and so adding to that very considerable number of lunatics, at least equally as numerous as those who at present find a refuge within the walls, quite unprovided for, and as official evidence proves, most injuriously and sadly placed for their interests and happiness. Therefore it is much to be wished that the prefect had propounded a third proposition—viz., to institute an additional asylum for chronic cases; for the two he has advanced will not lead to any diminution in the number of the insane population of his department, nor will their exclusion from an asylum prove an economical proceeding. On the contrary, they are both fallacious, if interpreted absolutely; for the determination betwixt dangerous and non-dangerous lunatics is practically not feasible, and the decision of the question of curability is very far from infallible.

The importance of early treatment is illustrated by the following results of statistics carefully kept for twelve years. Of patients deemed curable submitted to treatment in the first month of their disease, 66 per cent. recover; after the lapse of three months this proportion falls to 48 per cent., after six months to 40 per cent., after a year to 12 per cent., and beyond that period the falling off is still more decided. Relapses after recovery occur in the ratio of 28 per cent. The proportion of recoveries according to sex, is represented by the annexed figures:—of 1453 males treated, 309 recovered, or 24 per cent.; and of 1340 females, 376 recovered, or 29 per cent.; a difference between the two sexes explicable by the much greater frequency of paralysis and of the results of intemperance among males. The same causes likewise operate in causing an increased rate of mortality among male lunatics, which, during twenty years at Stephansfeld, has reached the high figure of 38 per cent., and in the case of the female inmates 31 per cent. Thus it appears that the mortality at Stephansfeld has equalled one-third of the whole population under treatment during the twenty years to which the statistics refer. The number of deaths relative to the population has varied materially in different years; on two occasions it reached 1 in 6, and on others it fell to 1 in 16. From 1836 to 1840, the rate of mortality was 1 in 8·9, or four times greater than in the neighbouring town of Brumath. Between 1841 and 1845 it decreased to 1 in 11; from 1846 to 1850 it stood at 1 in 10·7; and from 1851 to 1855 it was only 1 in 11. In 1847 the rate of mortality was very high, viz., 1 in 6; intermittent fever prevailed largely, and numerous cases of serious intestinal disorders, of dysentery, and of scurvy occurred.

In 1854, again, ague was very rife, owing to the earthworks for a new branch railway near the asylum, and cholera attacked sixty inmates, mostly on the female side, and carried off eleven women and five men. Lastly, although railway operations were completed, intermittent fever—with which 508 patients had been attacked in 1854—seized on 810 in 1855. This result was traced to the infiltration of the waters of the neighbouring canal into the basement of the building and to the consequent humidity of the asylum and neighbourhood. In May, 134 cases occurred, in June, 148, and in July, 128, after which the disease progressively declined.

Some German physicians have asserted that an attack of ague is curative of insanity in some instances. But this dictum is opposed to the large experience the Stephansfeld Asylum offered. In some rare cases, indeed, delirium abated during an intense febrile paroxysm, just as it is known to do under a variety of circumstances where a serious bodily affection is present; but as the disorder disappeared, delirium resumed its sway; moreover, most of the patients subjected to the influence of the marsh miasma had their vital powers reduced by it, and in such the mental affection acquired more or less speedily the stamp of incurability, and became transformed into dementia.

I am able to add the statistics of the admissions, discharges, and deaths, in 1856 and 1857:—

| Remaining on | Males. | Females. | Total. | Admissions in | Males. | Females. | Total. |
|---|---|---|---|---|---|---|---|
| Jan. 1st, 1856 | 288 | 308 | 596 | 1856 | 103 | 113 | 216 |
| Jan. 1st, 1857 | 291 | 324 | 615 | 1857 | 121 | 113 | 234 |

| Discharges in | Cured. | Relieved. | Unimproved. | Total. |
|---|---|---|---|---|
| 1856 | 38 | 65 | 22 | 125 |
| 1857 | 60 | 51 | 10 | 121 |

The deaths in 1856 amounted to 72; in 1857 to 93; being at the rate of 9 per cent. in the former, and of 11 per cent. in the latter year. In the spring of 1855, 27 general paralytics were under treatment; at the end of the year there were only 13; during 1856, 5 were admitted; and in the course of 1857, 27 were present, of whom 22 were men, and only 5 women. The epileptic insane in 1855 numbered 58—viz., 40 males and 18 females; during 1856, 53 were under treatment; and during 1857, 55, of whom 37 were males, and 18 females.

In the department of the "Bas-Rhin" the proportion of the insane to the population is calculated at 1 in 1320; whilst in that of the "Haut-Rhin" it is still lower, being only 1 in 1465. These figures are only approximative, for in some districts of each department no returns are made, and the departmental asylum is not resorted to for their lunatic inhabitants.

Numerous excellent comments on the etiology and pathology of insanity might be gathered from the painstaking reports of the Stephansfeld Asylum, drawn up by the physician, Dr. Dagonet, a gentleman particularly known for his earnest endeavours to cull facts from the large experience he enjoys in that institution. To those, therefore, of our readers who are interested in the annual record of the results of treatment which such an institution as Stephansfeld exhibits, we would commend the reports of Dr. Dagonet to their best attention. On our part, we are indebted to them for much of the foregoing statistical matters. Statisticians would likewise find in a little *brochure* published by him in 1855, entitled *Etude Statistique sur l'Aliénation Mentale dans le Department du Bas-Rhin*, an excellent investigation into the prevalence and etiology of insanity in the several arrondissements and cantons of that department, which might be advantageously adopted as a model for any similar inquiries instituted in this country.

Before taking leave of the statistics of Stephansfeld Asylum, one table merits the attention of every student of social science, as exhibiting not only the great proclivity of unmarried people to insanity, but also the extent to which celibacy must prevail in France. The table of the civil state of 849 inmates shows that the—

| | Total. | Men. | Women. |
|---|---|---|---|
| Unmarried amounted to . | 599 | 300 | 299 |
| Married . . . | 186 | 94 | 92 |
| Widowed . . . | 64 | 18 | 46 |
| | 849 | 412 | 437 |

The unmarried therefore constituted seven-tenths of the whole number in question; "a fortunate circumstance," rejoins M. Dagonet, "inasmuch as it is an obstacle to an augmentation of insanity by hereditary transmission."

M. Richard, who held the post of director of Stephansfeld since its occupation as an asylum, was replaced in 1859 by M. Brie de

Berc, who is a physician, and was transferred from his post at the asylum of Dijon. We hail this change at Stephansfeld from knowing that gentleman's earnest zeal and sound principles in the treatment of the insane, and we trust that M. Dagonet will find in him additional aid and support in every endeavour to ameliorate the condition of the asylum and its inmates.

There is yet one matter more connected with Stephansfeld which must not be passed unnoticed. It is the existence of a Benevolent Fund for the benefit of discharged patients, much on the same plan as that in operation at the Middlesex asylums, or as that recently originated by the Commissioners in Lunacy. It dates its foundation in the year 1842, and was the fruit especially of M. Richard's exertions, seconded by those of Dr. Roederer, the physician at that date. Since 1852, it has more directly received the encouragement and support of the departmental authorities, who have by a formal circular recommended its object to the mayors and other magistrates of the several towns and villages under their jurisdiction. In its scope it is more extensive than the similar English associations alluded to; it does not restrict its operation solely to a momentary relief of a discharged patient by the donation of a small sum in money, but it aims at establishing such an organization throughout the districts served by the Stephansfeld Asylum, that, when the patient is returned to his home and friends, he shall still be under the fostering care of benevolent persons, acting in co-operation with the officers of the Relief Fund and of the asylum.

The nature of this organization will be best illustrated by the published rules of the association :—

Art. I. The Benevolent Fund for the insane poor discharged cured from the asylum of Stephansfeld has for its general purpose the removal of the unhappy prejudice which prevails that insanity is an incurable disease, and for its particular objects :—

1. To protect and direct the poor of both sexes when discharged cured or relieved on their re-entrance into society.

2. To aid them according to their need by temporary relief in money or other things until they have regained confidence and found employment.

3. To secure on their behalf, in the localities they inhabit, the intellectual and moral support of enlightened and charitable persons so as to fortify them as far as possible against circumstances which might expose them to a relapse or to despondency.

Art. II. The members of the association are divided into three classes :—

1. Patrons or patronesses, who, besides an annual contribution to the fund of at least five francs each, undertake a personal supervision over the discharged patients, and distribute to their wants the aid awarded by the fund.

2. Subscribing associates, who, without taking personally any part in carrying out the details of the association, are willing to make a regular contribution to its resources.

3. Corresponding associates, who, without pledging themselves to make contributions to the fund, consent to directly protect and assist the discharged patients at their abodes.

Art. III. The management of the fund to be centralized at Stephansfeld, in the hands of a committee composed of the members of the asylum board of commissioners, of the director of the asylum, of the physician, the chaplains of the different religious persuasions, of the treasurer, and of the steward.

Art. IV. The president and vice-presidents of the asylum commission to hold the same offices in the association; the director and chief physician to be the secretaries; the treasurer to have the same office, and the steward to have charge, as storekeeper, of the effects belonging to the charity.

Art. V. The resources of the association are derived from: 1. Grants made by the superior authorities of the departments. 2. Subscriptions of the patrons and subscribing associates. 3. Voluntary donations. 4. The proceeds of lotteries, meetings, &c. 5. The proceeds derived from the sale of objects made by the patients or presented by other persons, for assistance upon their discharge.

Art. VI. The Treasurer to keep a ledger of all receipts and payments; not to retain in hand more than two, or at most three hundred francs; any excess to be deposited in the Strasburg Savings Bank so soon as it comes into his hands.

Art. VII. The director of the asylum may, in case of necessity, authorize the withdrawal from the savings bank of sums needed for the operation of the charity, provided that he informs the administrative committee of the circumstance at its next meeting.

Art. VIII. The deposit of the funds of the association in Government Securities, and the sale of such securities, not to be made except by the assent of the committee.

Art. IX. The expenses of the fund, and the grants in aid made to discharged patients, to be paid by the treasurer on a written order, signed conjointly by the director and the physician of the asylum, who are required to render an account of their proceedings to the administrative committee.

Art. X. The storekeeper to undertake to procure and to keep in stock goods of any description intended for discharged patients; to distribute such articles upon receiving a written authority signed by the director and physician of the asylum, and to keep a special account of the receipt and delivery of these articles.

Art. XI. The director of the asylum to present annually a summary of the proceedings of the past year, and a balance-sheet of receipts and expenditure. The physician at the same time to present a report on the results obtained by the charity in all that relates to the physical and moral health of the discharged patients.

Art. XII. The present regulations to be submitted for approval to the "Prefet du Bas-Rhin,"—in whom, we may add, the Benevolent Fund finds an energetic supporter, and who rejoices in the very un-Gallican, but yet very English name of West.

The circular addressed by the committee of the Benevolent Fund to the prefects, admirably points out the scope and utility of the charity. It remarks that, to guard against relapses so far as possible, it is not enough to present patients with a small gratuity on their discharge from the asylum, but that provision need be made to facilitate their obtaining employment, to surround them with a generous protection, and to secure for them the advice and aid of the industrial classes they are thrown among. It further points out that the districts to which the patients belong are directly interested in providing against their relapse; and yet that such poor persons are often abandoned to their own resources, without moral or medical supervision and succour, and are sent back to the asylum in a state of relapse, and without any information relative to the circumstances in which they have been placed during their absence.

Hence the committee recommend that the mayors and "district physicians" should watch over the interests of the discharged insane of their localities, and make reports from time to time to the asylum authorities of their physical and mental condition.

These recommendations of the committee have been ably seconded by the benevolent prefect of the Lower Rhine, M. West, who gave them the weight of his high official position by a circular addressed to all the mayors, district physicians, and ministers of religion in his populous department, wherein he lays down the following detailed plan for carrying them out by the co-operation of those civil and religious functionaries:—

The committee of the fund to address a circular, containing two schedules, to the mayor of the district to which the discharged patient belongs. The first schedule to contain—1, observations by the physician of the asylum on the nature and course of the mental affection for which the patient has been treated, and on the precautions necessary to be observed to avoid a relapse; 2, certain questions addressed to the district (cantonal) physician, and requiring replies from his own observation; 3, a place for the mayor of the commune to append information relative both in regard to the conduct and resources of the patient since his return home, and to the description of the extent and sort of aid which the Benevolent Fund should render him. This report should be filled up and sent in by the several mayors to the prefecture six weeks after the discharge of the patient. The second schedule to be similar, but not returnable until the expiration of six months.

The questions contained in the schedules refer to the conduct, moral and religious, of the patient, to the difficulties surrounding him, to the work he engages in and the manner in which it is performed, to the remuneration he obtains and to its sufficiency with or without a grant from the Benevolent Fund. Those more particularly addressed to the cantonal physicians inquire as to the persistence of the patient's recovery, to his relations with his family, to his occupation and habits, to any irregularities noticeable in his intellectual or moral perceptions, to any alteration or eccentricity of manner, and to his bodily health.

Although in this country we do not possess a social system like that of France, whereby every community and every individual is rendered part and parcel of the State machine, and can be subjected to oversight and control; although, therefore, we cannot transfer the organization of the benevolent institution under notice as an entirety among our own, yet we may derive some valuable suggestions from it calculated to improve and extend the operations of those useful funds connected with some English asylums for the relief of discharged patients. Voluntary association and co-operation take the place of State nurture and Government management in this free country where such charitable objects as the one in question are concerned, and I feel persuaded that it is only requisite to rouse the public mind to the necessity of taking some steps to uphold and protect the poor persons discharged from our lunatic asylums in their early struggles on readmission into the world, to secure an effective association and a sufficient fund. I would particularly call the attention of the Commissioners in Lunacy to the organization of the Stephansfeld Fund, since I find from their last report (13th) that they have started a fund for the relief of the same class of persons, and presume it is their intention to appeal to the public for co-operation and support. However, I am as yet in ignorance of the rules framed by the commissioners for its administration, and, indeed, whether they have as yet proceeded to frame any; still, in either case, the regulations of a similar charity will at least be interesting, if possibly not suggestive of improvements.

